# Smartphones as Catalysts for Synergistic Nutrition: A New Era in Bioactive Detection, Personalization, and Food System Intelligence

**DOI:** 10.1002/fsn3.70880

**Published:** 2025-09-02

**Authors:** Mohamed Ibrahim Younis, Yahia Ibrahim Sallam, Khaled Fahmy Mahmoud, Zheng Ruan, Rawaa H. Tlay, Tarek Gamal Abedelmaksoud

**Affiliations:** ^1^ Food Science Department Faculty of Agriculture, Cairo University Giza Egypt; ^2^ Food Technology Department National Research Center Giza Egypt; ^3^ State Key Laboratory of Food Science and Resources, Institute of Nutrition and School of Food Science Nanchang University Nanchang China; ^4^ Department of Food Science Faculty of Agriculture Engineering, Damascus University Damascus Syria

**Keywords:** artificial intelligence in nutrition, bioactive compounds, mobile spectroscopy, personalized dietary assessment, point‐of‐care food analysis, smartphone‐based sensing, synergistic nutrition

## Abstract

Naturally occurring bioactive compounds such as polyphenols, flavonoids, and vitamins play critical roles in human health and sustainable food systems. Yet their widespread utilization is constrained by complex detection methods and limited accessibility. This review explores how smartphones are emerging as transformative platforms for real‐time analysis, enhanced synergy discovery, and personalized nutrition. By integrating spectroscopy, imaging, electrochemical sensing, microfluidics, and AI, smartphones now enable field‐grade assays that rival laboratory precision at a fraction of the cost. Their deployment across agriculture, food processing, and consumer health is examined, with a focus on how smartphone‐based tools can be used to quantify synergistic interactions between bioactives, optimize nutrient retention, and deliver data‐driven dietary guidance. Coupled with machine learning, these devices can identify optimal compound pairings and adapt recommendations to individual physiology and environmental conditions. Limitations related to sensor calibration, data standards, and regulatory readiness are also highlighted. Finally, a roadmap for advancing smartphone‐enabled nutrition science through standardization, accessibility, and responsible innovation is presented. As smartphones evolve from passive sensors into intelligent, connected analyzers, they hold unprecedented potential to reshape food quality monitoring, democratize nutrition, and accelerate the global transition toward more resilient and health‐focused food systems.

## Graphical abstract

Smartphones are transforming nutrition science by enabling real‐time, low‐cost detection of bioactive compounds through integrated spectroscopy, sensing, and AI technologies. These portable tools support synergistic nutrient discovery, personalized dietary guidance, and intelligent food system monitoring. The study proposes a roadmap for standardization and innovation to maximize impact.

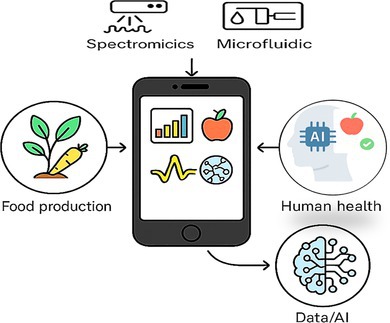

## 1 Introduction

Naturally occurring bioactive compounds—such as polyphenols, flavonoids, vitamins other phytochemicals in fruits, vegetables, and grains—are now recognized as vital to human health, nutrition, and disease prevention (Kussmann et al. 2023). These plant‐derived constituents, even at low levels, modulate metabolism, inflammation, and other physiological pathways, forming the molecular basis for diets that can prevent chronic diseases (from diabetes and obesity to cardiovascular and neurodegenerative disorders) (Deledda et al. 2021). Advances in genomics, metabolomics, and computational biology—exemplified by the rise of “Precision Nutrition” – are converging with medicine to harness bioactives for affordable, food‐based health solutions (Heber et al. 2023). This approach is not only crucial for human well‐being but also for planetary health: investing in bioactive‐rich foods and alternative natural ingredients (e.g., plant‐derived proteins, natural preservatives and soil‐enhancing biostimulants) can help feed a growing population more sustainably while protecting biodiversity and ecosystems (Galanakis 2024).

Despite this promise, realizing the potential of bioactive compounds is challenged by analytical limitations. Conventional detection and quantification methods (e.g., HPLC, mass spectrometry, spectrophotometry) are powerful but rely on expensive, bulky instruments and specialized laboratories. These assays are typically slow, require extensive sample preparation and expert operators, and are ill‐suited to field or point‐of‐consumption use (Behboudi et al. 2025). For example, standard tests for antioxidant or nutrient content in foods are time‐consuming and lab‐bound, making large‐scale or personalized monitoring impractical (Calabria et al. 2021). These barriers result in a lack of real‐time, accessible information on the actual bioactive composition of foods and diets around the world—representing a bottleneck for nutrition research, quality control, and preventative healthcare.

Recent years have seen smartphones emerge as transformative portable sensing platforms that can overcome many of these constraints. Modern phones combine high‐resolution cameras, powerful processors, connectivity, and even miniature optics, enabling them to serve as low‐cost analyzers (Hossain and Canning 2025). Researchers have already demonstrated smartphone‐integrated spectrometers and colorimetric readers that rival laboratory instruments. For instance, Kong et al. developed a CD‐spectrometer attachment for a phone that performed ultra‐portable, high‐sensitivity colorimetric assays—such as vitamin C (ascorbic acid) detection—with accuracy comparable to bench‐top spectrophotometers (Salinari et al. 2023). In general, smartphones offer key advantages over traditional optics: they are ubiquitous and affordable, bypassing much of the capital cost and technical complexity of lab devices (Blahnik and Schindelbeck 2021). Phones can directly image and analyze solid and liquid samples without cumbersome preparation, perform on‐device data processing, and instantly share results via networks—all of which greatly improve accessibility and speed (Upadhyay et al. 2024). In short, mobile‐phone platforms are disrupting bioanalytical chemistry by delivering lab‐quality detection at orders of magnitude lower cost and much higher portability.

An exciting frontier is the use of smartphones to probe combinations of bioactive compounds and the synergistic effects they may produce. It is well known that whole plant extracts often outperform isolated constituents precisely because of beneficial “phytosynergy” among their components (Caesar and Cech 2019). For example, complex herbal mixtures often demonstrate enhanced antioxidant or anti‐inflammatory activity compared to what would be expected if each component were acting independently, where interactions between compounds amplify their overall effect. Smartphone‐based sensors—especially those using multiplexed optical probes or arrays—could enable simultaneous, multi‐analyte measurements in food or biological samples. By capturing rich data on many compounds at once, these devices can help map out interaction landscapes of natural products. Coupled with computational analysis, mobile platforms may even facilitate the discovery of novel compound combinations. Looking ahead, the integration of smartphones with artificial intelligence is poised to accelerate this process: AI algorithms can mine large datasets of food composition to design new bioactive molecules or identify bioactive sequences in food proteins, while mobile apps deliver personalized dietary recommendations based on a user's unique physiology and food intake (Salinari et al. 2023).

Finally, smartphone‐enabled detection carries profound implications for sustainability, equity, and personalized nutrition. Low‐cost mobile sensors can democratize access to nutritional analysis, empowering farmers, producers, and consumers alike. For example, a grower could use a phone‐based device to measure antioxidant levels in crops on‐site, optimizing harvest and reducing waste (Saini et al. 2021). Similarly, smartphone apps can bring nutritional guidance to remote or underserved communities: by scanning food or test strips, individuals could receive tailored advice on diet quality or micronutrient status. This “citizen science” approach aligns with global goals of sustainable agriculture and public health—ensuring that nutrient‐rich, bioactive foods reach those who need them and that dietary interventions are informed by real data. As part of a broader vision, the synergy of mobile technology, cloud data, and omics promises a new era of nutrition science where every meal can be tracked, analyzed, and optimized for health. In sum, by transforming how natural bioactive compounds are detected and leveraged, smartphones may become pivotal tools in building a healthier, more sustainable food future.

## 2 Smartphone Technologies for Bioactive Compound Applications

Smartphones have emerged as versatile analytical platforms by virtue of their advanced optics, sensing, and computing capabilities (Bui et al. 2023). Modern phones integrate multi‐element camera modules, white‐LED flashes, touch screens, and a suite of environmental sensors (e.g., ambient light, proximity) in a handheld form factor (Bui et al. 2023). This rich hardware palette—combined with powerful processors and ubiquitous connectivity—enables smartphones to function as miniaturized laboratories for detecting bioactive chemicals. By capturing optical signals (colorimetry, fluorescence, etc.) and feeding them into onboard software, smartphones can effectively read out many assays that would traditionally require bulky instruments (Baker et al. 2025). In practice, the global ubiquity of smartphones (billions of users worldwide) makes them an ideal low‐cost platform for on‐site chemical sensing, especially in resource‐limited settings.

### 2.1 Hardware Innovations

Contemporary smartphones pack high‐resolution CMOS camera sensors with multi‐lens optics and digital image processing (Fang 2025). These cameras typically use Bayer color filters (RGGB) to capture visible light, and many phones now include multiple cameras with varying focal lengths. While color filters overlap spectrally, manufacturers mitigate low‐light limitations through pixel binning and added sensor modules (Pust 2021). For example, phones with quad‐Bayer filters automatically combine pixels to boost the signal at low illumination. Some handset cameras even use monochrome sensors (no color filters), which capture fluorescence signals at more than twice the intensity of standard color sensors (Pohanka 2020). Built‐in LED flashes (torch lights) provide approximately white illumination (blue LEDs with phosphor coatings) for exciting colorimetric or fluorescence assays (Fernandes et al. 2020). In short, the rapid evolution of smartphone optics—high frame rates (up to thousands of fps) and advanced light control—has greatly expanded their utility as portable detectors.

Beyond imaging, smartphones include other useful hardware. Ambient light and infrared sensors (originally for screen brightness and facial recognition) can double as simple photodetectors, while even the screen itself can serve as a multicolored light source for structured illumination. Crucially, the smartphone's battery and ports can power and control external devices. A modern phone's USB‐C port can supply ~5 V at ~2 A (≈10 W), sufficient to drive LEDs, lasers, or small pumps (Baker et al. 2025) (Older audio‐jack interfaces, where still present, have been used to power simple electrodes or read analog signals.) Additionally, Bluetooth, Wi‐Fi, and NFC radios provide wireless links to peripheral modules or cloud services (Boni et al. 2021). In practice, most smartphone‐based sensors leverage these interfaces to connect miniaturized hardware—such as microfluidic cartridges, electrochemical probes, or spectrometers—that clip on or snap into a custom housing. For instance, low‐cost clip‐on spectrometers or cuvette holders (often 3D‐printed) have been demonstrated for absorbance and fluorescence measurements, leveraging the phone's camera as the detector. Compact microscope attachments (some only a few grams and dollars in cost) have also been developed, enabling microscopy in the field (Salido et al. 2022).

Key hardware innovations include integration with microfluidics and printed electronics. Research groups have designed ‘lab‐on‐phone’ devices where samples flow through microfabricated channels under automated control. In such systems, the smartphone may simply image a reaction zone or provide timing and powering, while the microfluidic chip performs sample handling and mixing. These hybrid platforms exemplify the versatility of smartphone‐based hardware: by offloading assay chemistry to disposable chips, the smartphone remains the portable reader. Importantly, smartphone‐connected microcontrollers (e.g., Arduino‐compatible boards) and 3D‐printed components have driven down costs by orders of magnitude, turning complex lab setups into affordable point‐of‐use instruments (Umapathi et al. 2022). The bottom line is that off‐the‐shelf smartphone modules and affordable add‐ons can replace bench‐top spectrometers, potentiostats, or imagers in many applications, while offering true portability.

### 2.2 Software Innovations

On the software side, smartphones provide rich environments for data acquisition, processing, and user interaction. Custom mobile applications (apps) can control external hardware, capture images or sensor readings, and execute complex algorithms on the device. Many apps implement color calibration routines to convert raw RGB values from the camera into analyte concentrations, or to correct for ambient lighting variability. Other apps parse images of test strips or colorimetric reactions in real time, providing instant quantitative feedback on results. For example, a recent smartphone assay uses a phone camera to monitor solution color and convert it into RGB intensities for multiple pesticide residues (Tang et al. 2023). Similarly, specialized apps have been developed to automatically read lateral flow immunoassays: by analyzing the intensity of test and control lines on a strip, a smartphone can classify results as positive/negative much faster and more reliably than human eyes (Colombo et al. 2023). These computational methods often employ simple machine‐vision steps (edge detection, thresholding) to locate assay features, followed by statistical analysis to assign a result.

The incorporation of machine learning (ML) and artificial intelligence (AI) has further enhanced smartphone analytics. Convergent with advances in mobile AI, “smart” apps now exist that can identify complex patterns in sensor data. For instance, convolutional neural networks running on a smartphone have been used to classify cell images and flag pathology, or to recognize spectral signatures of analytes in diffuse samples. The promise is clear: as Baker et al. note, “smartphones with smart apps powered by machine learning… hold immense promise for realizing a future for molecular analysis that is powerful, versatile, democratized” (Baker et al. 2025). Even without ML, smartphones excel at multivariate processing: they can rapidly combine inputs from multiple sources (RGB channels, external sensors, geolocation) to distinguish subtle differences in analyte signals.

Connectivity and data sharing are also critical software assets. Smartphones can log results to cloud databases in real time, enabling longitudinal tracking or crowdsourced environmental monitoring. For example, GPS‐stamped measurements of water quality can be automatically mapped and shared, facilitating community science. Internet connectivity further allows smartphones to download reference libraries or lookup tables for compound identification, effectively augmenting the device's onboard analytical “library”. In field deployments, apps can include built‐in tutorials and decision support, guiding non‐specialists through sampling and interpretation—a key factor that makes smartphone assays accessible in low‐resource settings (Beduk et al. 2022).

Overall, software innovations leverage the phone's operating system and ecosystem. Modern smartphones have gigabytes of RAM and multi‐gigabyte storage, on‐chip GPUs for parallel processing, and powerful APIs (application programming interfaces) for camera and hardware control (Fabre et al. 2024). This means that smartphones can perform many calculations (including ML inference) locally, without needing a separate computer. Furthermore, the mobile app model enables rapid deployment of updates and new features. In short, the software suite of a smartphone—from image analysis libraries to networking protocols—transforms a bare‐bones sensor into a user‐friendly analytical instrument, with interactive graphics, data logging, and connectivity that traditional detectors lack.

### 2.3 Integrated Platforms and Applications

The synergy of smartphone hardware and software has spawned diverse real‐world applications. In environmental monitoring, smartphone‐based devices have been deployed for on‐site testing of water and soil. One notable example used a superhydrophobic “SPOT” concentrator with a smartphone camera and app to detect lead, nickel, chromium, copper, and cobalt ions simultaneously at ultratrace levels. This system could quantify multiple metals within 8 min at a cost of only $0.02 per test (Lee et al. 2021), illustrating both the speed and low cost of smartphone chemistry. In agriculture, handheld assays for pesticide residues have leveraged phones: a colorimetric sensor coupled to an app enabled on‐the‐spot quantification of organophosphorus pesticides by analyzing solution color changes (Tang et al. 2023). In healthcare, smartphone attachments and apps have been used for point‐of‐care diagnostics—for example, automatically reading rapid antigen or immunoassay strips for infectious diseases (Bermejo‐Peláez et al. 2022). In each case, the same underlying smartphone platform could be repurposed simply by changing reagents or software settings, demonstrating the device's versatility.

Importantly, these applications have been field‐tested in recent years (2020–2025) and even commercialized. Portable smartphone microscopes and spectrometers are already sold to field technicians, and dozens of research prototypes have progressed to pilot deployments in clinics, farms, and water utilities. The results emphasize the key advantages of smartphone platforms: they are truly portable (battery‐powered and compact), inexpensive (using mass‐market hardware and 3D‐printed accessories), and easy to operate by minimally trained users (Beduk et al. 2022). Because nearly every user carries a smartphone, widespread adoption does not require purchasing specialized readout units—one simply adds modular adapters or consumable test strips.

In summary, smartphone‐based sensing marries innovative hardware peripherals with powerful software apps to create analytical tools of remarkable flexibility. These systems can perform optical measurements (colorimetry, fluorescence, scattering) and even electrochemical assays through USB or Bluetooth probes, all interpreted by the phone's processors. The portability and low cost of smartphone platforms have already enabled applications from water safety to agricultural chemical monitoring (Haddout et al. 2024). Looking ahead, continued advances in camera sensitivity, microfluidic integration, and AI‐driven analysis promise to expand the range of detectable bioactive compounds. As one review observes, by transforming every smartphone into a potential laboratory, these technologies offer a “democratized” approach to molecular analysis that reaches far beyond traditional labs (Chakraborty 2024).

## 3 Smartphone‐Based Detection of Bioactive Compounds

Smartphones have rapidly emerged as portable analytical tools in chemistry and food science, capitalizing on their ubiquity and built‐in hardware. Modern phones combine high‐resolution cameras, powerful processors, wireless connectivity, and a user‐friendly interface into a compact device (Kalinowska et al. 2021). This integration allows a smartphone to serve as a controller, analyzer and display for chemical sensing, enabling “quick, authentic, and point‐of‐care” monitoring in contexts that would otherwise require a laboratory instrument (Kishore et al. 2022). As one review notes, smart devices could revolutionize “wide‐spread [food] quality assessment” from “farm to fork” by empowering non‐specialists to perform on‐site tests (Kalinowska et al. 2021). In practice, smartphones have been paired with simple consumables (test strips, dyes, nanoparticles) or miniaturized add‐on optics/electronics to detect nutrients, antioxidants and other bioactives in foods and plant samples. The result is a new class of low‐cost, portable assays that can run in the field or even at the point of consumption, where traditional lab equipment cannot easily reach (Vázquez et al. 2021).

### 3.1 Imaging and Spectroscopic Approaches

The most common smartphone assays use the built‐in camera to perform colorimetric or spectroscopic measurements. For example, by photographing a color‐changing test strip or solution under controlled lighting, a phone's image sensor can quantify an analyte through its effect on RGB color channels. In one notable study, researchers used a smartphone camera to measure the total phenolic content of vegetable oils. The phone's red‐channel signal correlated very closely with a standard UV–Vis spectrophotometer, yielding a “statistically equivalent” calibration (Vucane et al. 2024). In fact the authors described their smartphone method as a “superior alternative to traditional spectrophotometric methods,” achieving a low limit of detection (∼1.3 mg/L) and essentially matching laboratory precision. By exploiting simple color models and embedded image analysis, such camera‐based assays can be extremely fast (results in seconds to minutes) and avoid the need for bulky optics. Smartphones have also been combined with 3D‐printed “photo boxes” or external lenses to standardize illumination and improve repeatability, making the data more reproducible.

Beyond basic colorimetry, smartphone spectroscopy is rapidly advancing. With a small diffraction grating or prism placed over the phone's camera, one can build a miniature spectrometer: the camera image then records a dispersed light spectrum instead of a simple color. The phone's processing power can extract peak wavelengths and intensities in real time, effectively turning the device into a low‐cost visible or near‐infrared spectrophotometer (Huang et al. 2021). Such miniature spectrometers have been demonstrated to detect dyes, pigments and biomarkers in food and plant samples. Even Raman spectroscopy—a high‐end technique for identifying molecular fingerprints—has been adapted to smartphones. For example, engineers at Texas A&M developed a smartphone Raman system by coupling a laser and diffraction grating with the phone's camera. This setup could identify chemicals by their Raman spectra in the field, at far less cost and size than a conventional lab Raman instrument (Dhankhar et al. 2021). In their words, traditional Raman spectrometers are “large, expensive, and primarily confined to laboratory use,” whereas the smartphone version is much more compact and accessible. Other optical modalities (fluorescence, chemiluminescence) can be handled similarly: with appropriate external LEDs, filters or accessories, the camera can sense emitted light from fluorescent or luminescent assays. In short, smartphones are being turned into general‐purpose spectrometers and microscopes, leveraging their improving sensors and the creativity of phone accessory designers.

### 3.2 Integration With Chemical and Biosensors

In addition to purely optical methods, smartphones often serve as interfaces to chemical sensor chips and microfluidic devices. For instance, paper or plastic “dipsticks” with enzyme or nanomaterial coatings can produce a measurable color change in the presence of a target analyte; the smartphone simply reads and analyzes the resulting color or intensity. Electrochemical sensors—electrodes that generate a voltage or current in proportion to an analyte concentration—have also been miniaturized and paired with phones. In one design, a small potentiostat (or even a near‐field communication chip) connects wirelessly to a smartphone via Bluetooth or NFC, turning the phone into both the power source and display for the electrochemical measurement. Such smartphone‐controlled sensors have been built for water‐quality tests (nitrate/nitrite, heavy metals, toxins) and even metabolic assays. For example, an open‐source “universal wireless electrochemical detector” (UWED) was assembled from off‐the‐shelf components and paired with a smartphone. Because the UWED is “cost‐effective, compact, field‐portable, and completely wireless,” it is well‐suited to resource‐limited settings (Kishore et al. 2022; Wang et al. 2025). In practice, a smartphone can thus orchestrate a full analytical setup: it controls sample processing (microfluidic pumps or chemical reactions), reads signals (camera or electronic input), processes data, and maps or transmits results in real time.

### 3.3 Comparison With Conventional Methods

**FIGURE 1:**
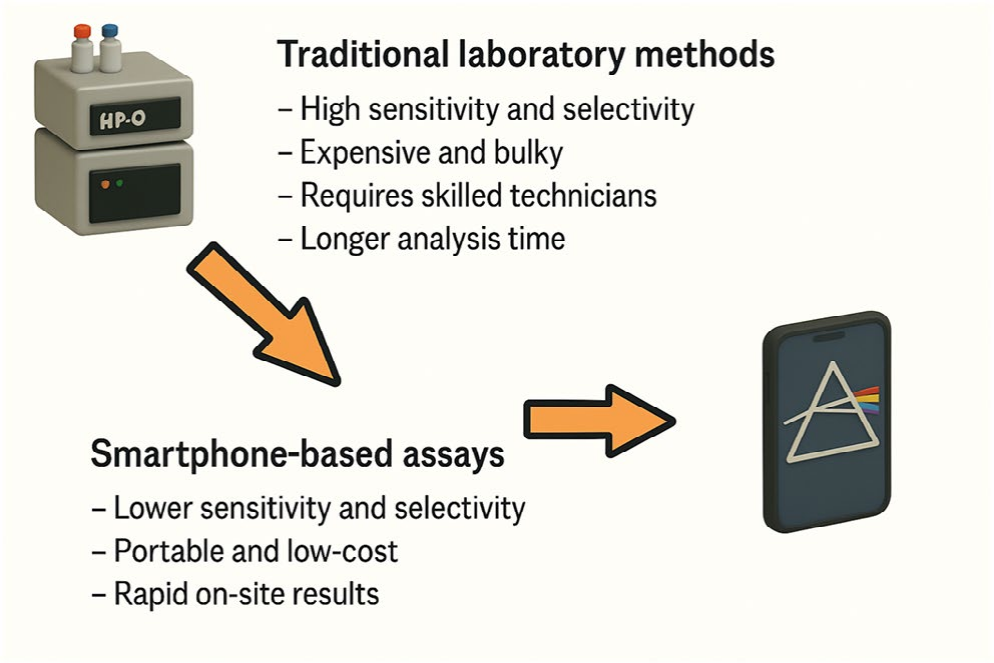
Smartphone‐based assays vs. traditional laboratory methods.

Smartphone‐based assays contrast with traditional laboratory techniques (HPLC, GC–MS, spectrophotometry, etc.) in several key ways (Figure 1). Conventional methods deliver the highest sensitivity and selectivity—they can resolve complex mixtures and detect compounds at very low levels. However, these instruments are costly (often tens or hundreds of thousands of dollars), bulky, and require skilled technicians and stable lab conditions. Systems like Raman spectrometers “require complex optics and high power” and are largely confined to the lab (Moon et al. 2023). In contrast, a smartphone‐based system is orders of magnitude cheaper (the phone is a standard consumer device) and can be deployed immediately in the field or at points of care. Speed is another advantage: a smartphone assay can give an answer in seconds or minutes on site, whereas sending samples to a central lab can take hours or days. Accessibility is similarly improved as nearly 90% of the world's population carries a smartphone (Senjam et al. 2021), so these methods enable decentralized testing outside urban centers or developed laboratories.

Of course, there are trade‐offs. Many phone‐based methods have somewhat lower precision or a narrower dynamic range than benchtop instruments, and they often require careful calibration and standardized lighting. Results can also vary between phone models, which use different cameras and default image processing. Nevertheless, published comparisons often show surprisingly good performance. In the vegetable‐oil phenolic example, the smartphone method matched the spectrophotometer well enough to report equivalent total phenolics within experimental error (Vucane et al. 2022). Similarly, smartphone chemometric tests have reached detection limits in the low micromolar range for many bioactives, approaching laboratory results. In some cases (e.g., simple color assays), smartphone readout can even improve on traditional visual scoring by providing objective digital data. Overall, smartphone devices tend to be faster, far more portable, and lower‐cost than HPLC or spectrometry, at the expense of some sensitivity and the need for robust calibration.

To fulfill their promise, smartphone assays must also overcome practical challenges. Recent reviews highlight that image analysis protocols and validation procedures are not yet standardized across devices (Maier‐Hein et al. 2024). Different phones and lighting setups can produce variability in the raw signals, so careful design (e.g., using calibration cards or fixed holders) is often required. Nevertheless, the gap is closing: as software improves and accessory modules become common (e.g., clip‐on spectrometer kits or fixed optical attachments), smartphone platforms are increasingly yielding reliable data. In many pilot studies, scientists have found the smartphone results “comparable and as accurate as those achieved through conventional analysis methods” (Straczkiewicz et al. 2021), especially for routine screening purposes.

## 4 Smartphone‐Assisted Enhancement of Bioactive Compound Utilization

Building on the advanced sensors, connectivity, and computing capabilities of modern smartphones, novel applications are emerging throughout the food system to not only detect but actively enhance the use of bioactive compounds in crops and foods. In agriculture, smartphones equipped with imaging, soil sensors, and AI‐powered apps enable precision farming that can maximize the nutritional quality of produce (Agrawal and Arafat 2024). In industrial processing, portable smartphone‐linked devices and IoT systems allow on‐site quality control and optimized extraction of phytochemicals. At the consumer level, mobile apps and “smart” kitchen tools guide diet personalization and food handling to preserve healthful compounds. Across all sectors, smartphone‐based sensing, data integration, and AI create feedback loops that improve identification, recovery, and stability of vitamins, antioxidants, and other bioactives, with important benefits for nutrition, health, system efficiency, and sustainability.

### 4.1 Agricultural and Primary Production Applications

Smartphones and allied technologies are increasingly used in the field to monitor and manage factors that affect bioactive compound content. For example, farmers use soil‐testing kits and connected sensors (moisture, NPK, pH) that interface with smartphone apps to map soil fertility across a farm. Machine‐learning models running in these apps can predict local nutrient status and recommend tailored fertilization and irrigation regimes. A recent mobile tool (“GeaGrow”) used artificial neural networks on smartphone data to give location‐based soil nutrient profiles and customized fertilizer plans (Folorunso et al. 2025). Such precision agronomy optimizes plant health and can increase yields of phytochemicals like flavonoids and vitamins, while using fewer resources. Similarly, smartphone cameras coupled with AI software are used to diagnose plant stress or deficiencies from leaf images. Deep‐learning apps can detect nutrient deficiencies or pests early from photos and prescribe corrective actions, helping to maintain or even boost levels of health‐promoting metabolites.

#### 4.1.1 Real‐Time Field Sensing

Mobile soil‐ and crop‐sensing devices connected to smartphones provide on‐site data (e.g., soil moisture or chlorophyll indices) that farmers use to adjust inputs. For instance, petiole and leaf greenness meters link via Bluetooth to mobile apps to measure chlorophyll content, a proxy for plant nutrient status. This allows targeted fertilization so crops accumulate optimal levels of micronutrients and antioxidants (Saiz‐Rubio and Rovira‐Más 2020; Soussi et al. 2024).

#### 4.1.2 Data‐Driven Recommendations

AI‐powered smartphone apps combine satellite/drone imagery and field sensor data to generate precise crop protocols. One example is the GeaGrow app, which provides smallholder farmers with personalized, location‐specific fertilizer recommendations based on predicted soil NPK and organic matter (Folorunso et al. 2025). By adapting inputs to real conditions, such tools improve the efficiency of nutrient use and can enhance the synthesis of bioactive compounds (e.g., certain stress‐regulated flavonoids) in fruits and vegetables (El‐Ramady et al. 2022).

#### 4.1.3 Optimized Harvest Timing

**FIGURE 2:**
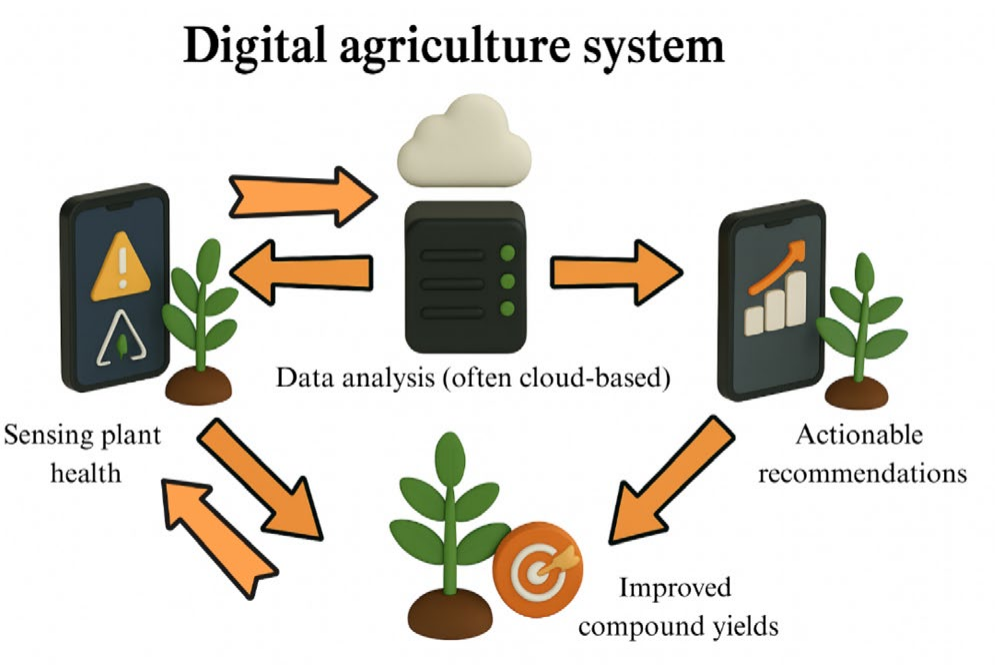
Closed‐loop “digital agriculture” system.

Mobile imaging is also used to time harvests for maximal bioactive content. Smartphone apps can analyze fruit color or firmness to predict ripeness and polyphenol levels, ensuring that produce is picked at peak nutritional quality (Sherafati et al. 2022). In high‐value horticulture, smartphone‐linked refractometers or NIR devices help determine sugar and vitamin content in real time, guiding both harvest and post‐harvest handling to preserve antioxidants. These smartphone‐enabled practices create a closed‐loop “digital agriculture” system (Figure 2).

Overall, smart agricultural tools reduce wasted inputs (fertilizer, water) and can drive sustainable intensification: healthier plants with richer phytonutrient profiles are grown on the same or fewer acres.

### 4.2 Industrial Processing and Quality Control Applications

In food processing and manufacturing, smartphones are transforming quality control and extraction methods to preserve and quantify bioactive compounds. Portable smartphone‐based assays allow on‐site testing of antioxidants and other phytochemicals in raw materials and products, reducing reliance on distant labs. For instance, researchers developed a 3D‐printed microchip system paired with a smartphone camera to measure total antioxidant capacity (via gold‐nanoparticle colorimetric reaction) in teas, juices, and even olive oil (Calabria et al. 2021). The cheap, disposable cartridges and LED scanner produced results comparable to conventional assays [], enabling manufacturers to rapidly screen batches for polyphenol or vitamin content during production. Similarly, a handheld device using a flexible microchip and Wi‐Fi scanner was created for on‐site quantitation of anthocyanins in fruit beverages (Salimi et al. 2021). Images of the chip, captured by a smartphone app, yielded anthocyanin concentrations on par with laboratory spectrophotometry. Such mobile systems empower processors to monitor extraction efficiency and adjust parameters (e.g., solvent pH, time) in real time to maximize yields of colorants and antioxidants.

Smartphone technologies also integrate into larger IoT quality‐control networks. Advanced packaging concepts embed biosensors that change color when spoilage or oxidation begins. A consumer or processor can scan a package with a phone to read a freshness indicator or verify that bioactive‐rich ingredients are still potent. In one review of smart packaging, for example, biosensors enabled real‐time spoilage detection while IoT connectivity (often interfacing via smartphones) enhanced traceability (Sobhan et al. 2025). Such systems help identify substandard lots before they move down the line, cutting waste. In fermentation and processing plants, smartphone‐linked sensors can also log temperature, pH, or oxygen levels during production; AI routines then analyze these data to predict the retention of heat‐sensitive nutrients and adjust conditions accordingly.

#### 4.2.1 On‐Site Chemical Assays

Smartphones serve as portable spectrometers and colorimeters. For example, a smartphone camera combined with a calibrated light source was used to perform spectrophotometric analysis of vitamins and other micronutrients in food extracts. In a recent prototype, a smartphone spectrophotometer quantified vitamin B12 in a sample with over 90% accuracy (Balch et al. 2024). Translated to industrial settings, such devices could verify the concentrations of added vitamins or natural bioactive extracts (like carotenoids) in processed foods.

#### 4.2.2 Image‐Based Analysis and QC

Machine‐vision apps on smartphones can inspect product color, turbidity, or texture—proxies for bioactive content. For instance, smartphones can estimate chlorophyll or carotenoid levels in oils by analyzing their hue, providing quick checks on olive oil quality (de Carvalho and Nunes 2021). Similarly, cameras track the color change of enzymatic browning in fruits to ensure gentle processing. When integrated with AI, these image analyses become predictive: apps trained on large datasets can forecast shelf life or nutrient decay, warning operators to modify packaging or formulations.

#### 4.2.3 Data Integration and Waste Reduction

Beyond individual sensors, smartphones aggregate data into cloud platforms for big data optimization. By logging quality metrics and production parameters centrally, companies use AI to identify inefficiencies in preserving bioactives. For example, linking smartphone‐collected QC data with supply‐chain information helps trace sources of nutrient loss (e.g., slow chilling of berries). In smart factories, mobile dashboards can alert managers to adjust workflows (such as reducing heat exposure time) to improve the overall yield of functional compounds. The net effect is greater food system efficiency: less raw material is wasted, and final products deliver higher antioxidant or vitamin levels per unit input (Bhatlawande et al. 2024).

### 4.3 Consumer and Nutritional Applications

For end consumers, smartphones are becoming personal nutrition assistants that encourage the use and retention of dietary bioactives. Nutrition and diet apps leverage food databases, image recognition, and AI to help users choose and prepare foods rich in beneficial compounds. For example, an AI‐based nutrition app generated meal plans with significantly higher micronutrient content than users' usual diets (Hinojosa‐Nogueira et al. 2023). By recommending menus dense in vitamins, minerals, and plant polyphenols, such apps can steer consumers toward eating more bioactive‐rich fruits, vegetables, and whole grains. Food scanning apps (e.g., barcode or ingredient scanners) alert shoppers to products' nutrient profiles and can even highlight those with extra‐added antioxidants or superfoods. The smartphone thus acts as an interface for “precision nutrition,” translating individual health data and preferences into dietary guidance that maximizes bioactive intake.

Smartphones also help preserve nutrients in food at home. Mobile apps like the USDA's FoodKeeper teach users how to store foods under ideal conditions. For instance, the FoodKeeper app “maximizes the freshness and quality” of items by recommending optimal refrigeration and storage times (Patra et al. 2022). Following these guidelines helps maintain vitamins and phytonutrients that would otherwise degrade. Likewise, smart kitchen appliances controlled by smartphones—such as sous‐vide circulators or low‐heat steamers—can be programmed to cook foods gently, retaining heat‐sensitive nutrients (e.g., vitamin C, lycopene) that are often lost in conventional cooking.

#### 4.3.1 Personalized Diet Tracking

Mobile diet‐tracking apps use photo logging and AI to estimate nutrient intake and suggest improvements. Some apps can recognize foods from a photo and calculate their nutrient content, including antioxidants. Combined with wearable devices, smartphones can monitor biomarkers (like blood glucose) and correlate them with dietary bioactive intake, enabling feedback loops for healthier eating. Research shows that users of technology‐assisted dietary advice can achieve better micronutrient balance: one study found that AI‐generated menus had less variability and higher concentrations of micronutrients than participants' usual diets (Kaya Kaçar et al. 2025).

#### 4.3.2 At‐Home Testing and Education

Simple smartphone‐based test kits allow consumers to check food quality themselves. For example, paper‐strip assays with color‐change reagents (for vitamin C or antioxidant activity) can be read by a smartphone app. Educational AR (augmented reality) apps can overlay nutritional info or antioxidant values on foods when viewed through the phone camera (Chai et al. 2022). These tools raise awareness of bioactive content. In addition, by scanning “smart” food packaging sensors or QR codes, consumers can access freshness data (e.g., remaining shelf life or nutrient degradation alerts) at the grocery or home, promoting timely consumption before nutrients are lost.

Overall, smartphone‐enabled consumer tools help connect daily behavior with nutritional outcomes. By making the value of bioactive compounds visible and actionable, these technologies encourage diets richer in antioxidants and vitamins—with positive implications for long‐term health.

### 4.4 Integration and Implications

Across agriculture, industry, and households, smartphones serve as hubs that collect, analyze, and act on data related to bioactive compounds. Crucially, the integration of data streams (crop sensor outputs, production logs, consumer habits) through cloud platforms and AI creates a digital thread from farm to fork. For example, farm app data on soil nutrients can be linked with processing records on phenolic content to refine cultivar selection; likewise, consumer usage patterns can feed back to industry R&D on preservative methods. This cyber‐physical linkage ensures that knowledge about bioactive utilization accumulates and improves over time.

The implications are far‐reaching. Nutritionally, these technologies can help ensure that plant‐derived vitamins, polyphenols, and other healthful compounds are maximally retained from field to plate, supporting better human health. Economically and environmentally, precision tools reduce waste and input usage: IoT‐enabled packaging with smartphone interfaces cuts spoilage (Trejo Beltran 2023), and targeted farming lowers fertilizer runoff. Personalized nutrition platforms driven by smartphone AI may help prevent micronutrient deficiencies by nudging consumers toward balanced, nutrient‐dense diets (Rojanaphan 2024). In sum, the smartphone's sensing, data, and AI capabilities are transforming the food system into a more efficient and sustainable network for delivering bioactive‐rich foods—closing the loop between detection and enhanced utilization of nature's nutritional compounds.

## 5 Smartphone‐Assisted Synergistic Effects Between Bioactive Compounds

**FIGURE 3:**
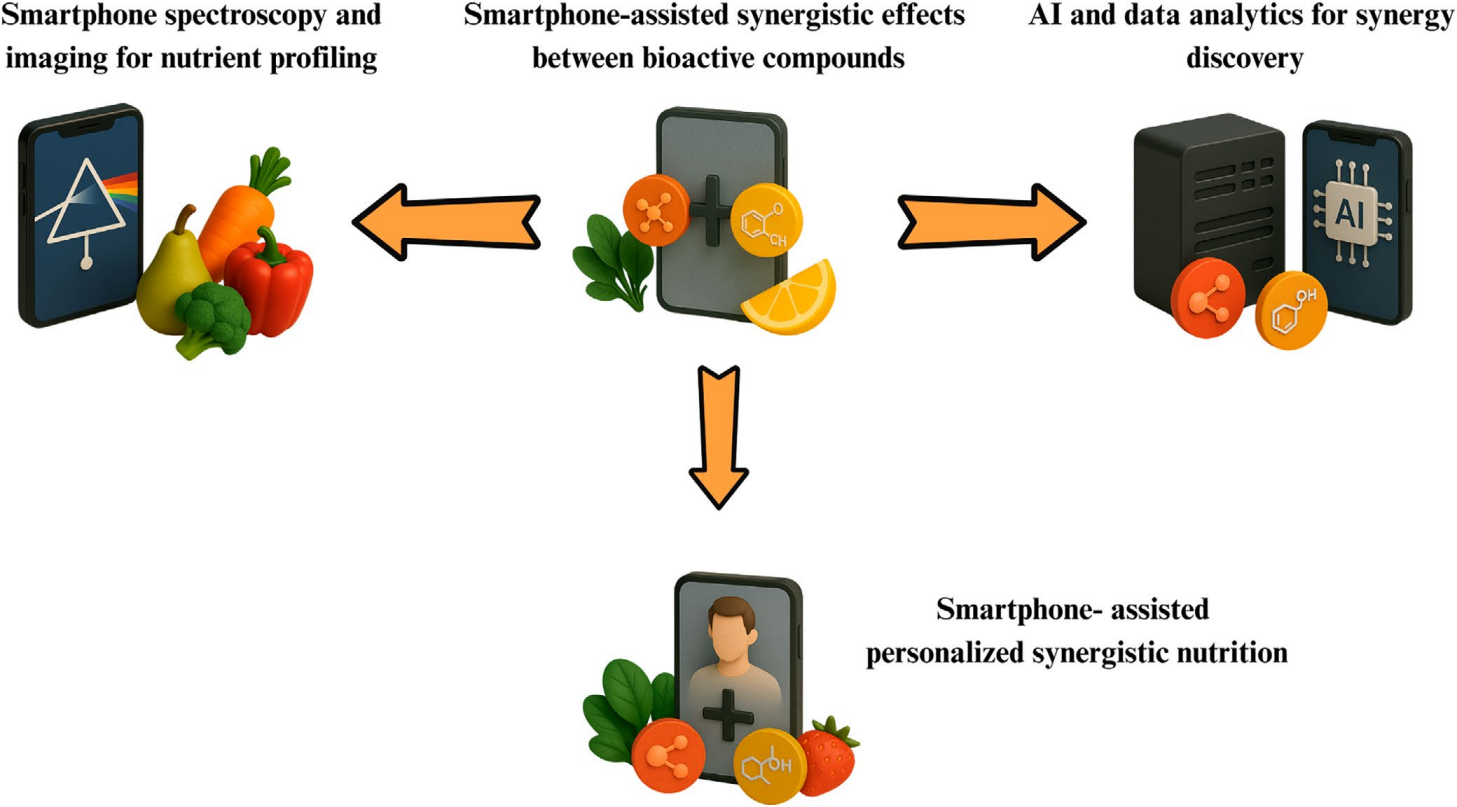
Smartphone‐centered framework for discovering and delivering personalized synergy.

Nutrient synergy refers to the phenomenon that combinations of bioactive food compounds can elicit greater biological effects than expected from their individual contributions (Townsend et al. 2023). New technologies now offer unprecedented tools to uncover and harness such synergy in complex foods (Figure 3). In particular, modern smartphones—equipped with cameras, sensors, connectivity, and AI—are being repurposed as portable chemical analyzers and data hubs. Handheld spectroscopy and imaging apps can detect dozens of compounds in fruit, juices, or spices, while machine‐learning analytics can model their interactions. Together, these tools can reveal multi‐compound “cocktails” in food that boost nutrient stability, bioavailability, or health impact.

### 5.1 Smartphone Spectroscopy and Imaging for Nutrient Profiling

Researchers have shown that mobile phones can serve as compact spectrometers or colorimetric scanners to fingerprint food compositions. For example, a custom smartphone spectrometer captured visible‐range spectra of Schisandra fruit extracts and, using built‐in chemometric analysis, correctly distinguished geographic origin as well as traditional NMR metabolomics (Kwon et al. 2016). Similarly, near‐infrared and even simple RGB imaging with a phone camera have been used to predict anthocyanin and total polyphenol levels of berries and juices. In one study, partial‐least‐squares models trained on ninety‐six açai samples linked NIR spectra or smartphone images to reference antioxidant metrics (total anthocyanins, polyphenols, ORAC) (Caramês et al. 2021). Although NIR hardware showed higher sensitivity, the smartphone‐based models still achieved useful accuracy (*R* ^2^ ∼0.7) for rapid “in situ” screening of fruit bioactivity.

Other portable devices convert color changes or fluorescence into chemical readings on a phone. For instance, a 3D‐printed smartphone chemosensor exploited gold‐nanoparticle formation: antioxidants in a tea or oil sample reduced gold (III) in a hydrogel, producing a color signal captured by the phone's camera (Calabria et al. 2021). This device delivered total antioxidant capacity (TAC) values for teas, herbal infusions, and olive oil that closely matched standard ORAC assays. In another advance, researchers demonstrated a wireless smartphone spectrometer (88 × 37 × 22 mm) with UV excitation and fluorescence detection. The handheld unit, complete with an on‐phone app, measured chlorophyll fluorescence of apples and correlated well with fruit ripeness (Das et al. 2016). These examples highlight that smartphones can now serve as low‐cost, portable labs—combining illumination, spectral filtering, imaging, and data processing—to detect multiple nutritional compounds simultaneously. Such multi‐wavelength sensing is a critical enabler for detecting interactions among bioactives: by characterizing the combined chemical fingerprint of two foods, smartphones can quantify how mixtures differ from the sum of parts.

### 5.2 AI and Data Analytics for Synergy Discovery

Beyond raw sensing, AI and computational models are poised to discover novel synergistic combinations. Food chemistry and health data can be cast as networks that link ingredients to molecular components and biological effects. For example, Veselkov et al. used network‐based machine learning to map almost 8000 food‐derived molecules and identified dozens of “cancer‐beating” bioactives by similarity to drug action profiles (Veselkov et al. 2019). Similarly, the FlavorGraph approach encoded foods and 1500 flavor compounds as a large graph, then learned embeddings that cluster foods with similar chemistry and suggest complementary pairings (Park et al. 2021). These graph and embedding techniques—powered by big data and AI—can be extended to model functional synergy rather than just taste. In principle, one could train a machine‐learning model (e.g., a deep neural network or graph convolution) on datasets of combined food ingredients and measured health readouts (antioxidant output, nutrient uptake, metabolic impact). Smartphone apps and cloud platforms could then use such models to predict which food‐pair “profiles” will produce enhanced bioavailability or biological effects.

Today's smartphone users are already generating relevant data: image‐analysis apps for meal recognition, portable metabolite sensors, and even citizen‐science food diaries. This crowd‐sourced data, coupled with AI, might identify unexpected synergistic patterns (Alamri 2024). For instance, adding citrus juice to a berry extract maximizes antioxidant yield, or certain herb‐spice mixes supercharge nutrient absorption. Moreover, a priori computational tools can guide targeted experiments. Virtual screening across large libraries of phytochemicals could flag pairs that operate on complementary pathways (e.g., one antioxidant that regenerates another's active form). Smartphones fitted with microfluidic chips or mini‐spectrometers could then validate these predictions in real food samples on‐the‐fly. In this way, mobile AI blurs the line between field analysis and lab research, enabling dynamic discovery of bioactive synergies.

### 5.3 Case Examples: Nutrient Synergy in Foods

Several well‐studied examples illustrate how compound combinations can boost nutritional effects and how mobile technology can study them (Table 1). One classic case is the interaction of polyphenolic flavonoids with vitamin C. Plant flavonoids (e.g., quercetin, catechins) act as antioxidants and can “spare” ascorbate from oxidation, effectively increasing vitamin C's stability in food and in vivo (Carr and Vissers 2013). In vitro studies even show curcumin (a polyphenol from turmeric) and quercetin working synergistically with ascorbic acid to inhibit protein aggregation, an effect greater than either component alone (Jahić Mujkić et al. 2021). On an analytic front, a smartphone spectrophotometer or colorimetric kit could simultaneously track both vitamin C and flavonoid signals in a beverage, revealing enhanced overall radical‐scavenging capacity in mixtures of berry (rich in flavonoids) and citrus (rich in vitamin C).

**TABLE 1:** Synergistic bioactive compound combinations: Detection methods and mechanisms.

Compound pair	Source foods/ingredients	Observed/measured synergistic effect	Detection method	Proposed mechanism of interaction	References
EGCG (tea catechin) + Vitamin C	Green tea + citrus (lemon/orange)	Enhanced antioxidant capacity and stability (EGCG degradation attenuated by Vit C)	Smartphone‐assisted AuNP colorimetry (total antioxidant capacity)	Vitamin C regenerates oxidized EGCG radicals	Calabria et al. (2021); Messire et al. (2023)
Anthocyanins + Gingerols	Berries (e.g., blueberry) + ginger	Strong synergistic enhancement of cellular antioxidant activity (↑CAA, ↓ROS) and cytoprotection	Cellular antioxidant assay (fluorescent ROS probe)	Preservation/upregulation of SOD, GPx and glutathione levels	Abdurrahim et al. (2023)
*Prosopis cineraria* + *Mangifera indica*	Desert legume (Prosopis) + mango fruit	Synergistic DPPH•‐radical scavenging (mixture EC_50_ ≪ individual EC_50_)	DPPH/FRAP spectrophotometric assays (portable/smartphone)	Combined pool of diverse phenolics (LC–MS found ~43 compounds)	Joshi et al. (2024)
Curcumin + Vitamin C	Turmeric (curcumin) + citrus (Vit C)	Synergistic inhibition of amyloid fibril formation (stefin B) in vitro	Thioflavin‐T fluorescence assay (portable reader)	Cooperative radical‐scavenging (Vit C may regenerate oxidized curcumin)	Jahić Mujkić et al. (2021)
EGCG + Kaempferol	Green tea + kale/onions (kaempferol)	Synergistic cellular antioxidant protection (↓ROS, ↑SOD/CAT/GSH‐Px)	Cell‐based ROS assay (fluorescent dyes)	Upregulation of antioxidant enzymes (SOD, CAT, GSH‐Px)	Zhang et al. (2023)

Another example is the famous spice–fruit synergy: adding black pepper (piperine) dramatically boosts the bioavailability of curcumin from turmeric. Co‐administration studies report a ~20‐fold increase in curcumin absorption in humans and animals when taken with piperine (Prasad et al. 2014). A smartphone‐enabled assay could exploit this by measuring curcumin concentration or its fluorescent metabolites in real‐time after ingestion of different food formulations. On the kitchen lab bench, a user might scan a homemade curry (imaging its color or analyzing a small extract) and, with a trained AI app, predict the enhanced curcumin content due to pepper. Similarly, smartphone NIR imaging has been used to quantify anthocyanins and polyphenols in mixed fruit purées, offering a way to test whether blending certain berries and herbs yields a higher antioxidant index.

#### 5.3.1 Other Synergistic Trends Can Be Explored With These Tools

For instance, combining vitamin‐rich foods (citrus fruits) with fiber and polyphenol‐rich ones (apples, berries) to improve micronutrient absorption or modulate the gut microbiome. Mobile spectroscopy can measure related markers such as sugar, flavonoids, and even fermentation products (Franca and Oliveira 2021; Pan et al. 2024). Likewise, coupled smartphone‐wearable platforms might monitor postprandial blood glucose and GI hormones, linking them back to meals analyzed by phone—effectively profiling how food combinations impact metabolic responses. In essence, every smartphone assay reading of a multi‐ingredient sample provides data on the emergent effect of those combined bioactives.

### 5.4 Future Directions: Personalized Synergistic Nutrition

Looking ahead, the marriage of smartphone sensing and AI promises personalized synergy optimization (Heber and Li 2024; Tiwari and Waoo 2024). By continuously capturing individual dietary intake (via food‐logging apps or image analysis) and even biological feedback (e.g., wearable glucose or blood markers), smartphones can feed personal “nutrition omics” data into on‐device AI. These algorithms could then recommend not just nutrient intakes but also synergistic pairings tailored to the user's genetics, microbiome, and health goals. For example, an app might identify that a person's low vitamin D uptake is enhanced when consumed with certain fats or flavonoids, and suggest recipe tweaks accordingly.

In food product development and supply chains, similar principles apply. Manufacturers could use smartphone‐enabled spectrometers on the production line to verify that a new fruit blend truly contains the expected cocktail of phytonutrients in effective ratios. Custom AI models could then tweak formulations (e.g., adjusting spice blends or fortification levels) to maximize functional synergy. Ultimately, as mobile sensors and AI mature, a Synergy App Store is envisioned: a suite of smartphone tools designed to analyze meals in real time and provide advice on next steps (e.g., eat an orange, add basil) to unlock specific health benefits.

In summary, smartphone‐based platforms are transforming how compound synergies in foods are detected and designed. By coupling multi‐spectral sensing (spectroscopy, imaging, chemometrics) with machine learning, researchers and consumers alike can probe the hidden interactions among vitamins, polyphenols, and other phytochemicals. This approach elevates nutrition science from single‐nutrient focus to a holistic view of food matrices. As these technologies converge, they open the way to intelligent, synergy‐aware nutrition—where every meal can be analyzed and optimized for maximal health impact, right from your phone. Key applications of smartphone‐assisted nutrient synergy:

#### 5.4.1 Multi‐Compound Sensing

Portable phone spectrometers and imaging analyze complex mixtures of flavonoids, carotenoids, vitamins, etc., enabling on‐site assessment of combined antioxidant or bioactive content (Caramês et al. 2021; Moscetti et al. 2025).

#### 5.4.2 AI‐Driven Pairing

Machine‐learning models (e.g., graph embeddings) learn chemical relationships to suggest complementary ingredients and predict emergent bioactivity (Periwal et al. 2022; Zhang et al. 2024).

#### 5.4.3 Customized Formulations

On‐phone apps and cloud analytics tailor food pairings to individual profiles, guiding recipe choices or product design for enhanced nutrient synergy (Turkay et al. 2024).

#### 5.4.4 Health Monitoring

Integrated sensors (camera, wearables, ingestibles) track the physiological outcomes of food combinations, closing the loop from chemical profile to wellness effect (Alam et al. 2025; Coman et al. 2024).

These developments foreshadow a future of personalized synergy nutrition, where what is eaten is not only tracked by smartphones but also actively shaped by them—unlocking new levels of health benefit from the foods already consumed.

## 6 Challenges and Limitations

### 6.1 Technical Constraints

**FIGURE 4:**
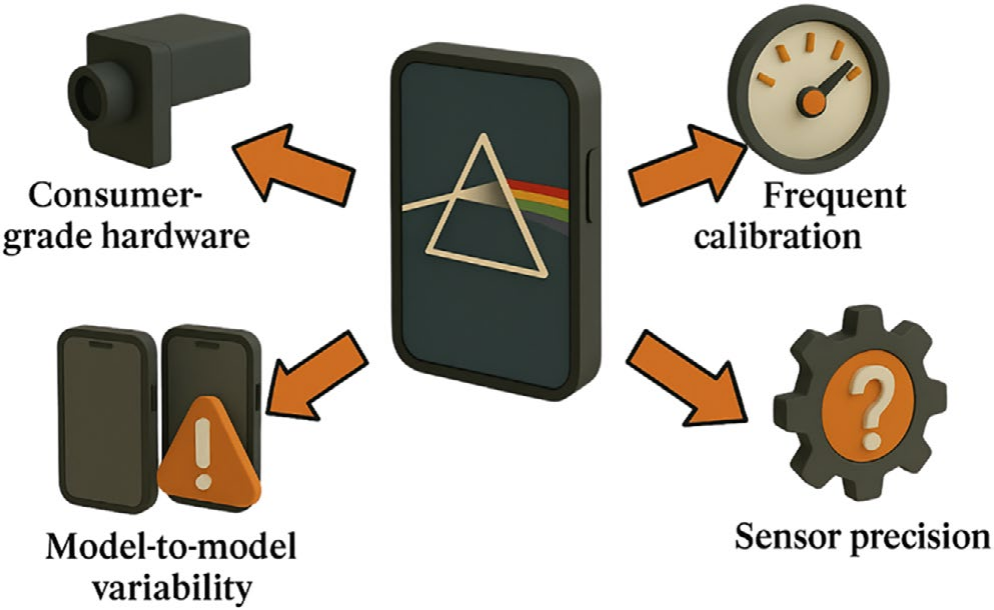
Technical limitations of smartphone‐based sensors.

Smartphone‐based sensors are inherently limited by consumer‐grade hardware (Figure 4). Camera optics and sensors are designed for general photography, not precision spectroscopy, leading to issues with resolution, sensitivity and calibration. For example, add‐on spectrometers have achieved ~15 nm wavelength resolution, but a 10‐bit analog‐to‐digital converter (ADC) in a microcontroller (versus 16‐bit in lab spectrometers) restricted the intensity resolution, reducing sensitivity to small signal changes 84. The narrow field‐of‐view and lens aberrations of phone cameras also constrain achievable resolution (often ∼1 μm in prototype microscopes). In practice, ensuring reproducible measurements across models is very challenging. Different smartphone models have varying image sensors, optics and automatic settings, making it “impractical to adjust every assay to every smartphone model” (Bui et al. 2023). Consumers are typically reluctant to attach external optics or illumination, so any additional hardware must be minimal (Blahnik and Schindelbeck 2021). Moreover, smartphone spectrometers and fluorescence readers usually require careful calibration. Calibration typically relies on external reference sources (e.g., lamps or dyes) and must be repeated whenever the device is opened or moved, since even small temperature changes or mechanical shifts can cause spectral drift (Markvart et al. 2020). Some recent work has proposed “smart” assays and apps that self‐calibrate or flag inconsistent results to compensate for device variation (Baker et al. 2025), but practical solutions are still under development. Until standardized calibration protocols and hardware are established, sensor precision will remain a bottleneck.

### 6.2 Analytical and Sample‐Related Challenges

Food and plant matrices are highly complex, often containing many colored, scattering, or fluorescent constituents. Only about half of reported smartphone assays have been validated on real foods (Ji et al. 2020; Nelis et al. 2020); the rest use simple water samples or extracts, reflecting how sample complexity can undermine performance. Foods typically include pigments (chlorophyll, carotenoids, polyphenols, etc.) that interfere with optical assays. Such color interferences raise detection limits or shorten linear ranges in colorimetric tests. For example, the green or red hues in fruits and vegetables can obscure subtle color changes from target analytes. Likewise, turbidity and autofluorescence in opaque or solid samples can mask analytical signals (Baker et al. 2025; Peveler and Algar 2021). In practice, many smartphone methods rely on laborious sample preparation to mitigate these issues: filtration of particulates, centrifugation to separate phases, or chemical extraction of analytes. These steps require trained users and bench equipment, negating much of the promised “field” convenience. In effect, the “world‐to‐chip” bottleneck persists: very few devices perform a true sample‐to‐answer test. Indeed, only ∼17% of published methods process raw samples directly on‐device (Baker et al. 2025; Khosla et al. 2022). Overcoming food‐matrix challenges will demand innovative chemistries and microfluidics (e.g., chromogenic reagents or micro‐total‐analysis‐systems) that can selectively react with targets in crude samples, or digital processing (e.g., background subtraction) to account for variable matrix color (Hernández‐Mesa and García‐Campaña 2020). Until then, complex matrices remain a major obstacle to accuracy and reliability.

### 6.3 Data and Machine Learning Limitations

Many smartphone assays now incorporate machine learning (ML) for signal analysis or interpretation, but these models face their own limits. Successful ML requires large, diverse training datasets that cover the full range of sample and device variability. Unfortunately, there are few standardized spectral or image databases of food bioactive compounds. Existing food composition databases emphasize macronutrients and common micronutrients; dedicated databases for diverse phytochemicals are still under development (Durazzo and Lucarini 2021). The incompleteness of bioactive compound data means that ML models are often trained on narrow sets of examples. As a result, models can overfit to specific lighting conditions, phone models, or sample types and may perform poorly on new datasets. In effect, the generalizability of ML‐based detection is unproven. Moreover, smartphone image channels (RGB) are an imperfect proxy for continuous spectra, so many papers rely on calibration curves or simple color ratios (Fan et al. 2021). Future progress will require open, curated repositories of annotated reference spectra or images for key bioactives, as well as domain adaptation strategies. Until ML models can be rigorously validated against standardized benchmarks across labs, their scientific reliability and regulatory acceptance will remain uncertain.

### 6.4 Infrastructural and Social Barriers

**FIGURE 5:**
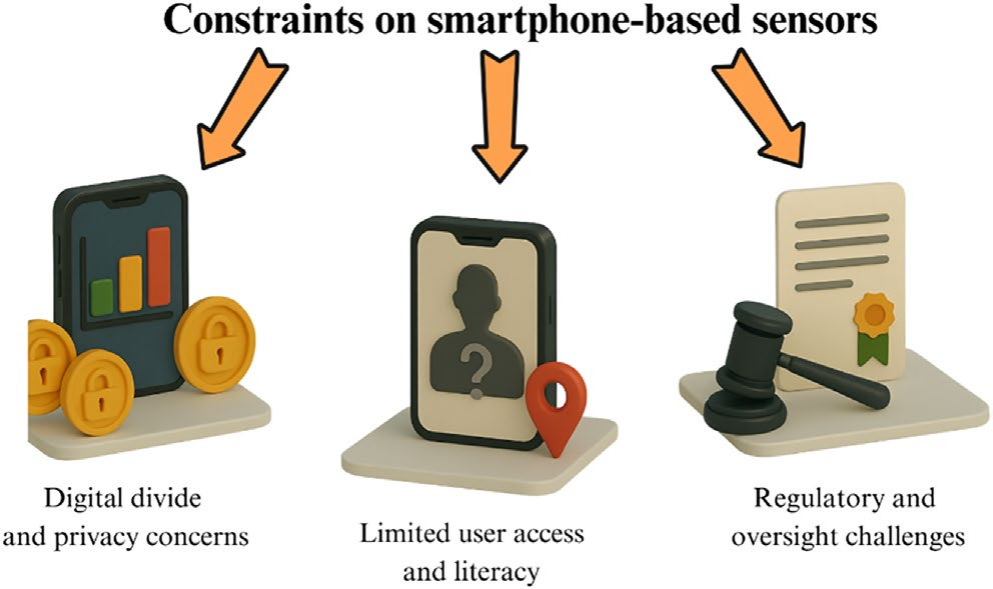
Infrastructural and social barriers concerning smartphone‐based sensors.

Even a technically robust smartphone sensor can falter without the proper infrastructure and user base (Figure 5). First, access and literacy are uneven. Global data show that only ~63% of people have internet connectivity, and in the poorest countries that drops to ~27% 93. Smartphone penetration and network coverage vary widely; rural or low‐income communities—where food safety issues can be severe—are often the least connected. In addition, many end‐users (e.g., smallholder farmers or consumers) lack training in analytical procedures or data interpretation. A device that requires precise pipetting or image framing may be misused. Data privacy is another concern: smartphone assays often collect personal or location‐linked data (dietary habits, health markers, geotagged results). This raises questions of consent and security, similar to mobile health apps (Jacobson et al. 2020). Smartphones can store sensitive biometric or genetic information, and their small size makes them easy to lose or steal (Hamidi 2019). Robust encryption and privacy safeguards must be built in, yet regulations in this area are still emerging. Finally, regulatory frameworks lag behind technology. A food‐contaminant test on a phone might fall under the purview of food safety agencies or even medical‐device regulators, depending on claims. As one analysis noted, regulatory approval is likely a greater barrier than technical security issues (Smyth 2020). In sum, the digital divide, privacy concerns, and unclear oversight all constrain uptake: widespread adoption will require policy development, user training, and affordable, accessible devices that meet local needs.

### 6.5 Ethical and Environmental Considerations

The shift to consumer electronics for food analysis also brings ethical and sustainability questions. On one hand, empowering citizens to test their food could democratize safety monitoring. On the other hand, there is potential for misuse or misinterpretation. Over‐reliance on smartphone readings might give users a false sense of certainty or lead to unnecessary alarms. Data gathered by apps (e.g., diet or supplement intake, or biomarker levels) could inadvertently be sold or exposed, raising ethical red flags (Rothstein et al. 2020). E‐waste is a serious environmental issue as well. Globally, ~62 million tonnes of electronic waste were generated in 2022, yet only ~22% was formally recycled. Discarded phones and sensors release toxic materials into soil and water if not properly handled. Promoting smartphone‐based testing may accelerate device turnover unless sustainable design and recycling are enforced. Responsible development must therefore balance technological benefits against potential environmental harm and ensure data are handled ethically.

### 6.6 Prototypes and Commercialization Gaps

Despite extensive research, relatively few smartphone‐based assays have reached commercial markets. Analyses in the biosensor field observe that “smart biosensors on the commercial stage are still scarce” despite rapid academic progress (Madrid et al. 2022). A similar gap exists in food analysis. Many published prototypes rely on custom 3D‐printed holders, lab reagents or bespoke software—impractical for mass production. Achieving consistent performance across unstandardized consumer phones is also a hurdle for manufacturers. Moreover, transitioning from prototype to product requires regulatory clearance (often a multi‐year process for diagnostic devices) and user acceptance. In healthcare contexts, one proposed pathway is to certify specific phone+attachment combinations for professional use, but no such pathways currently exist for food‐testing apps. Until industry and regulators collaborate on standards (e.g., defining accuracy requirements or calibration protocols for mobile food sensors), smartphone assays will remain largely in the research domain.

### 6.7 Open Challenges and Outlook

Many challenges must be addressed before smartphone‐based bioactive sensing can become reliable and mainstream.

#### 6.7.1 Standardization and Calibration

There is a critical need for standardized protocols (e.g., fixed light sources, reference targets, or software controls) to ensure that measurements are comparable across devices. Future designs may include built‐in calibration references or use of invariant assay zones, and algorithms that auto‐correct for phone‐to‐phone variability (Rochi et al. 2024).

#### 6.7.2 Comprehensive Databases

Expanding and harmonizing databases of food bioactive compounds and their optical signatures is essential. Such resources would enable robust model training and cross‐study comparisons, similar to efforts in human metabolomics (Durazzo and Lucarini 2021).

#### 6.7.3 Advanced Data Analysis

Improving ML and AI methods (including explainable models) to handle smartphone data streams is a priority. Models must be trained on diverse, representative datasets and continually validated. Approaches like physics‐informed ML or transfer learning might help models generalize despite varying hardware and light conditions.

#### 6.7.4 Validation and Regulation

Field trials and round‐robin studies are needed to quantify how smartphone assay performance compares to gold‐standard lab methods. Engagement with regulators (food and health agencies) will help define acceptable use cases (e.g., preliminary screening vs. confirmation tests). In the near term, sensors may be cleared for limited purposes (e.g., screening in low‐resource settings) with the understanding that confirmatory lab tests are required for critical decisions.

#### 6.7.5 Accessibility and Ethics

Any deployment strategy must consider the digital divide: developers should aim for low‐cost solutions and offline functionality. Clear privacy policies and user consent protocols are necessary to ethically manage personal data. Recycling programs and eco‐design can mitigate environmental impact as the technology scales (Ratner et al. 2020).

Another important challenge is making smartphone apps that have easy‐to‐use user interfaces and data visualization tools. These platforms want to communicate complicated nutritional analyses to a wide range of people, including consumers and non‐specialists. To do this, they will need displays that are clear, interesting, and aware of the context. Future app development should use design principles that focus on the user and use visual metaphors, simple dashboards, and guided interpretation features to help people understand technical data in a way that they can act on it.

Addressing these open issues will require interdisciplinary collaboration between engineers, chemists, data scientists, policymakers, and end‐users. Only through concerted efforts on hardware, software, and policy fronts can smartphone‐based detection of food bioactives achieve the robustness and trustworthiness needed for broad scientific and societal adoption.

## 7 Future Trends and Perspectives

Smartphone‐connected sensor networks exemplify the emerging mobile bio‐sensing paradigm. Smartphones now serve as hubs for miniaturized assays and networked detectors: for example, phone‐driven microfluidic devices can perform field‐grade ELISA tests (Chen et al. 2014), and graphene‐based nanomaterial electrodes promise ultrasensitive detection of antioxidants and other bioactive compounds (Sainz‐Urruela et al. 2021). Combined with powerful cameras and processors, these tools effectively turn pocket devices into portable biochemistry labs capable of analyzing food phytochemicals on the spot. Such platforms leverage the ubiquity and connectivity of phones—low‐cost hardware that can geolocate a sample and instantly send results to the cloud (Broekman and Gräbe 2021). Mobile phone instrumentation has advanced dramatically in the past decade: novel on‐phone sensors detect an ever‐increasing range of analytes (Kishore et al. 2022), from light and color changes to chemical signatures, making smartphones versatile general‐purpose biosensing platforms. These trends point toward a future where everyday devices not only capture images but also actively interface with nanotechnology‐enhanced sensors and lab‐on‐chip modules to profile nutrients and contaminants in foods and plant products.

Wearable and real‐time biosensing technologies will further extend this mobile sensing revolution. Future wearables may embed hybrid nanosensor arrays that continuously monitor multiple biomarkers—for instance, glucose, hydration, and metabolic byproducts in sweat or interstitial fluid (Vo and Trinh 2024). Advances in energy harvesting and biofuel cells will enable these sensors to be self‐powered, eliminating batteries and allowing continuous data collection around the clock. Smart fabrics or patches could integrate seamlessly into clothing or accessories, turning garments into health‐tracking platforms. Crucially, these devices will stream data via Bluetooth or NFC to smartphones, which aggregate and analyze the information. In turn, users could receive real‐time feedback or alerts (e.g., nutrient deficiencies or glycemic excursions) based on sophisticated algorithms. Realizing this vision will require robust privacy protections and new regulatory frameworks. Guidelines for hybrid sensors and implantables must be established to build user trust and ensure safety (Arandia et al. 2022).

At the same time, artificial intelligence and big data analytics will transform how mobile sensing data are interpreted in nutrition and health. Machine learning models can ingest the vast streams of data generated by smartphone apps and wearables—including dietary logs, activity levels, and environmental measures—to deliver personalized nutrition advice. Recent analyses identify AI applications spanning personalized diet planning, image‐based food recognition, and predictive modeling of nutrition‐linked diseases (Theodore Armand et al. 2024). The integration of these technologies holds great promise: one study concludes that “AI, machine learning, and big data are a boon to the goal of sustainable food security” by enabling more efficient, data‐driven decision‐making (Namkhah et al. 2023). Indeed, the proliferation of food‐related data presents a major opportunity: as food and health data are increasingly collected through mobile platforms, they can reveal new insights to optimize food quality and public health (Biermann et al. 2021). Pioneering efforts have already begun to apply these tools for sustainability: for example, leveraging sensor data to improve agricultural productivity or using aggregated nutrition data to refine dietary guidelines. As the EAT‐Lancet Commission has noted, addressing food‐system issues is arguably the most powerful lever to improve both human health and environmental sustainability globally. In this new paradigm, smartphones and AI form the digital nervous system of food‐health systems, linking individual nutrient exposure to larger epidemiological and ecological models.

Crucially, these technological advances must translate into broader societal and public health benefits. Mobile biosensors could democratize access to nutrition information at unprecedented scale. With roughly 76% of people in developed economies (and a growing fraction in developing countries) owning smartphones (Hicks et al. 2023), low‐cost mobile assays can reach underserved populations for whom laboratory testing is inaccessible. Citizen‐science models will be powerful here: researchers note that “the ubiquitous presence of smartphones allows researchers to leverage citizen‐owned…devices as mobile data collection tools” (Katapally et al. 2018). For instance, community members might use smartphone apps to crowdsource data on food nutrient levels or contaminant presence, creating real‐time maps of nutritional status. Smartphones already empower individuals to screen food and water safety—recent researches highlight their potential to “revolutionize food safety control by empowering citizens to perform screening tests” (Chen et al. 2024; Nelis et al. 2020). Aggregating such participatory data could alert public health officials to nutritional deficiencies or foodborne hazards faster than ever before. On the individual side, mobile platforms can deliver tailored guidance: by combining personal sensor readings with AI analysis, apps could offer customized diet recommendations and early‐warning health alerts, ushering in an era of precision nutrition.

Moving forward, realizing this vision will demand concerted interdisciplinary effort and supportive policy. Research must focus on translating lab prototypes into rugged, user‐friendly tools: for example, developing standardized calibration methods for smartphone assays, open data formats for sensor outputs, and interfaces designed around user needs. The industry can help by mass‐producing affordable sensor attachments and embedding analytics into consumer apps. Importantly, regulators and policymakers have a role to play. Standards and validation protocols will be needed to ensure that mobile nutrient tests are reliable and comparable. Data governance frameworks must safeguard privacy while allowing data sharing for the public good. Guidelines for the safety and efficacy of mobile health devices should evolve as technologies mature; one perspective stresses that “regulatory frameworks should change accordingly” to match wearable biosensor innovations (Karim 2021). Finally, equity must be a priority: efforts should aim to bridge the nutrition gap, for example by incentivizing deployment of mobile nutrition tools in low‐income and rural communities. In sum, by integrating mobile sensing, AI, and sustainability thinking, a new data‐driven paradigm for food and health can be built. Such convergence promises to transform nutritional science and food systems—but it will only succeed through cross‐disciplinary collaboration and policies that align technology with public health goals (Hicks et al. 2023).

## 8 Conclusion

The convergence of smartphone technologies, chemical sensing, and artificial intelligence is redefining how bioactive compounds in foods and plants are detected, analyzed, and utilized. What was once the domain of specialized laboratories—quantifying polyphenols, vitamins, flavonoids, and other health‐promoting molecules—is now increasingly accessible through portable, affordable, and user‐friendly smartphone‐based platforms. These systems integrate optical detection, microfluidics, electrochemical sensors, and machine learning algorithms to deliver laboratory‐like functionality in the palm of a user's hand. Across agriculture, industry, and nutrition, the implications are profound. In food production, smartphones enable real‐time, in‐field monitoring of crop quality and harvesting decisions. In processing environments, they support point‐of‐line quality control for bioactive retention, minimizing waste and optimizing functional food formulations. At the consumer level, mobile apps and sensing kits can help individuals evaluate food quality, improve dietary decisions, and track bioactive intake, ushering in new models of personalized and precision nutrition. Moreover, the ability to uncover and enhance synergistic interactions between compounds—previously difficult to study outside the lab—now becomes possible through on‐device data integration and AI‐driven analysis. Yet while the potential is vast, several critical challenges remain. Calibration variability across devices, analytical interferences in complex food matrices, and limited access to standardized datasets all hamper scientific rigor and widespread deployment. Societal barriers such as digital inequity, regulatory ambiguity, and environmental concerns related to e‐waste must also be addressed to ensure these technologies deliver equitable and sustainable benefits. Moving forward, concerted efforts will be needed across disciplines to build interoperable standards, ethical frameworks, and robust validation pipelines that elevate smartphone‐based sensing from promising prototypes to globally impactful tools. As smartphones evolve into platforms for sensing, computation, and decision‐making, they will not only augment scientific workflows but also democratize access to nutrition intelligence worldwide. From farmer to food technologist to individual consumer, mobile sensing systems offer a new model for enhancing the reach, responsiveness, and precision of health‐promoting food systems. In this transformation, smartphones become not just communication tools but catalytic instruments for a more transparent, equitable, and bioactive‐aware future in nutrition and health.

## Author Contributions

**Mohamed Ibrahim Younis:** writing – original draft, formal analysis, data curation, visualization, methodology, conceptualization, software, writing – review and editing. **Yahia Ibrahim Sallam:** formal analysis, data curation, visualization, methodology, conceptualization, writing – review and editing, supervision. **Khaled Fahmy Mahmoud:** methodology, conceptualization, writing – review and editing, supervision. **Zheng Ruan:** methodology, conceptualization, writing – review and editing, supervision. **Rawaa H. Tlay:** writing – original draft, formal analysis, data curation, visualization, methodology, conceptualization, software, resources, writing – review and editing, supervision. **Tarek Gamal Abedelmaksoud:** writing – original draft, formal analysis, data curation, visualization, methodology, conceptualization, software, writing – review and editing, supervision.

## Conflicts of Interest

The authors declare no conflicts of interest.

## Data Availability

Data will be made available on request.
